# A plausible role of imagination in pretend play, counterfactual reasoning, and executive functions

**DOI:** 10.1111/bjop.12650

**Published:** 2023-04-03

**Authors:** Gill Althia Francis, Jenny Louise Gibson

**Affiliations:** ^1^ Department of Education University of York York YO10 5DD UK; ^2^ Faculty of Education University of Cambridge Cambridge CB2 8PQ UK

**Keywords:** counterfactual reasoning, executive functions, hypothetical model, imagination, pretend play

## Abstract

A notable observation is the similarities in the cognitive processes of pretend play (PP) and counterfactual reasoning (CFR) as both involve thinking about alternatives to reality. It is argued by Weisberg and Gopnik (*Cogn. Sci.*, *37*, 2013, 1368) that alternative thinking in PP and CFR is underpinned by an imaginary representational capacity but few studies have empirically investigated this link. We use a variable latent modelling approach to test a hypothetical model of the structural relationship of PP and CFR predicting that if PP and CFR are cognitively similar; they should have similar patterns of associations with Executive Functions (EFs). Data were collected on PP, CFR, EFs and Language from 189 children (*M* = 4.8 years, males = 101, females = 88). Confirmatory factor analyses showed that measures of PP and CFR loaded onto single latent constructs and were significantly correlated (*r* = .51, *p* = .001) with each other. Hierarchical multiple regression analyses revealed that EF accounted for unique significant variance in both PP (*β* = 21) and CFR (*β* = 22). The results of the structural equation modelling revealed that the data were a good fit for the hypothetical model. We discuss the plausible role of a general underlying imaginative representational capacity to explain similarities in the cognitive mechanisms of different states of alternative thinking like PP and CFR.

## INTRODUCTION

A particularly intriguing observation is the stark similarities in descriptions of the cognitive processes that facilitate pretend play (PP) and counterfactual reasoning (CFR). Although the two are seemingly distinct, the cognitive mechanisms through which they are activated bear a marked resemblance. According to Weisberg and Gopnik (2013), both PP and CFR involve ‘disengaging with current reality, making inferences about the events and scenarios that would follow in an alternate reality, and keeping these alternative possibilities separate from reality’ (p. 1370). So, how is it that a child pretending that a ‘banana is a telephone’ or an adult imagining that ‘if they left home earlier, they would not have missed the train’ involve similar mental processes (Byrne, 2016; Leslie, 1987)? Weisberg and Gopnik (2013) posited that since an imaginary representation of the world that is contrary to reality is evoked in both contexts, PP and CFR likely engage the same component cognitive abilities. Drawing on Weisberg and Gopnik's work, this study examined arguments that a general underlying imaginary representational capacity underpins associations shared by PP and CFR. We designed a testable hypothetical model of how PP and CFR are likely associated and generated empirical evidence to test the model. To begin, we critically review the literature about the cognitive mechanisms of PP and CFR and consider arguments about how the two are related.

### The cognitive mechanisms of pretend play

‘Pretend Play’, ‘make believe’, ‘fantasy play’, ‘symbolic play’ and ‘acting as if’ all denote pretend play (PP). In PP non‐literal representations of the world or an alternative world, which at the time is not real, are imagined. The consensus is that PP emerges from 18 to 24 months (Piaget, 1962; Thompson & Goldstein, 2019; Weisberg, 2015). It becomes consolidated into a child's play repertoire by their third year of development and by the fourth year, children's pretence capacities evolve to support the creation of elaborate fantasies that involve imaginary characters and animals (Nielsen & Dissanayake, 2000; Woolley, 1995). Pretend play declines after the middle childhood period, although people continue to have an appreciation for the imaginary as adults (Smith, 2009). Between the ages of 3 and 5, children begin to make clear reality and non‐reality distinctions of pretence and understand that in comparison to knowledge, imagination reflects reality less accurately (Weisberg, 2013; Woolley, 1995). On one hand, it is contended that children struggle to understand that pretence involves mental representation (Lillard, 1993), whereas, on the other hand, Woolley (1995) argues that children can actually understand mental representations in pretence if the tasks are simplified such that the linguistic demands are minimized and the salience of actions and mental state actions are balanced. Sobel and Lillard (2001) suggested that when the pretence involves fantasy characters children's understanding of the mind may be more advanced. In sum, children's ability to recognize and take part in pretence is independent of (and precedes) their capacity to understand the concept of pretence as a mental state (Smith, 2002). After reviewing over 50 years of PP research Thompson and Goldstein (2019) suggested that PP behaviours are likely to develop additively from least to most psychologically complex progressing from an ability for object substitutions, attributing pretend properties to objects, social interactions within pretend, role enactment, to pretence‐related metacommunication.

Perhaps the most influential account of the cognitive mechanisms of PP is put forward in Alan Leslie's ‘decoupling model of PP theory’ (Leslie, 1987). According to Leslie, through perceptual processing children take in information about their current experiences, which are processed as primary representations of the world. In PP, this primary representation (real‐world mental model) is raised to a second‐order representation or metarepresentation (mental model of the pretend world). Inference rules and other information are applied to decouple (the creation of a copy of primary representations) and quarantine primary representations from second‐order representations (Leslie, 1987). Quarantine refers to pretend representation (second‐order or metarepresentations) being marked off from primary representations (real‐world representations) such that the dual representations are held in mind separately without confusing the two (Leslie, 1987). The distinguishing skill that facilitates successful pretending is the absence of ‘representational abuse’ as children can maintain a distinction between PP and reality such that children hold both mental models in mind to draw upon appropriately as needed – either during PP or when navigating real‐world contexts.

### The cognitive mechanisms of counterfactual reasoning

Counterfactuals are diverse and can range from imagined alternatives that entertain and amuse, such as those found in fantasy, fiction or theatre to imagined alternatives that support logical, mathematical and scientific reasoning (Byrne, 2016). During CFR, mental representations that are explicitly contrary to facts or beliefs are imagined (Roese & Morrison, 2009). More precise definitions focus on mentally travelling back in time to observe something that actually happened and imagining a change to what actually happened by simulating how this alternatively would have turned out differently (Beck, Riggs, & Burns, 2011; Gerstenberg, 2022; Rafetseder et al., 2013). The question of whether children can reason counterfactually and what types of thinking count as true CFR is frequently debated in the developmental literature (Beck, Riggs, & Burns, 2011; Nyhout & Ganea, 2019a; Rafetseder et al., 2013; Rafetseder & Perner, 2010, 2014). A perspective, argued by Beck, Riggs, and Burns (2011), is that the development of CFR from early to middle childhood is not simply one critical development rather it broadly follows a pattern of at least four sequences of hypothetical thinking – the ability to create alternative worlds, represent falsity as if it were true, represent multiple possibilities, and compare multiple possibilities.

There are distinct cognitive features that mark each developmental phase of CFR. Early indicators include the ability to create alternative worlds including pretend play, an understanding that something ‘almost’ happened, and thinking about future hypotheticals (Beck & Guthrie, 2011; Beck, Riggs, & Burns, 2011; Harris, 1997; Robinson & Beck, 2000). By the age of three children can tell what actually happened in relation to what is imagined, what might have happened and what could happen indicating that young children have an understanding that observed outcomes (actualities) might have turned out differently (Harris, 2000). However, a criticism is that these early indicators of CFR do not involve the key counterfactual feature of negating what one knows to be true (Beck, Riggs, & Burns, 2011; Perner et al., 2004; Robinson & Beck, 2000). By age four, children can represent falsity as true which means that they can reason from a false premise (Beck, Carroll, et al., 2011; Beck, Riggs, & Burns, 2011), but responses are closely coupled to alternatives that are plausible real‐world answers – basic conditional reasoning. Rafetseder and Perner (2010, 2014) found that basic conditional reasoning leads to different answers from CFR and children are successful at CFR by the age of six. The cognitive feat is to overcome the nearest possible world constraints, that is to hold counterfactual possibilities and the actual possibilities (dual possibilities) in mind in the way that adults do (Beck, Riggs, & Burns, 2011). Recent research, however, contends that 4–5 years old can demonstrate mature CFR using a physical causal task (Nyhout & Ganea, 2019b). The final ability children develop is an understanding of counterfactual emotions, by age five and older (Beck et al., 2006; Beck, Riggs, & Burns, 2011; McCormack et al., 2018; Rafetseder et al., 2013; Rafetseder & Perner, 2014). By this point, children are successful at mature CFR and can hold multiple possibilities in mind by making comparisons between reality and what could have happened. A similar developmental trajectory is described in a review by Gautam et al. (2019). They explain that children's development typically proceeds in three stages: ‘(1) the capacity to imagine and reflect on affirmed and uncertain past, present, and future outcomes, (2) the capacity to imagine and reflect on counterfactual, negated versions of known past outcomes and present situations, and (3) the capacity to anticipate experiencing counterfactual emotions (i.e., regret and relief) in the future' (p. 1).

An explanation of the cognitive mechanisms of CFR is posited by Byrne (2016) as involving mental representations and cognitive processes that (a) take as input the relevant facts of actual events (grounded in reality); (b) produce as output a counterfactual alternative (an alternative to reality); (c) change aspects of the mental representation of the facts via intervening processes to create a second mental representation – the counterfactual alternative; and (d) have as a goal to produce counterfactuals that are plausible, that is reasonable, believable and acceptable. Therefore, the underlying mechanisms of CFR maintain and update two representations, the imagined alternative and the known or presupposed reality (Byrne, 2016). This makes the counterfactual thought dynamic as it can be challenged and changed by the discovery of further information.

### Comparing the cognitive mechanisms of pretend play and counterfactual reasoning

**FIGURE 1:**
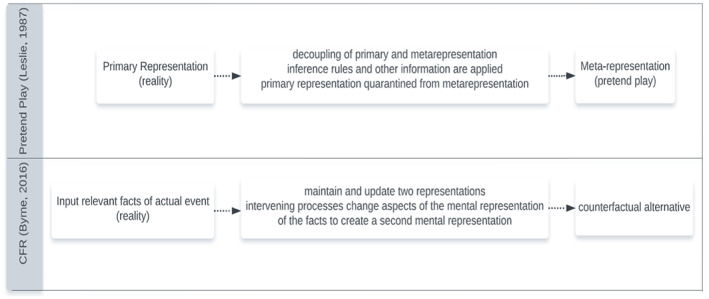
Comparison of the cognitive mechanisms of PP and CFR.

The cognitive mechanisms of PP and CFR proposed by Leslie (1987) and Byrne (2016) are compared in Figure 1. Both PP and CFR theorists make clear that alternative representations are imaginary representations derived from real‐world premises. Leslie (1987) clarified that the mental representations that underpin our PP are drawn from our perceptual processes, and Byrne (2016) proposed that during CFR, we take as input the relevant facts of actual events. Additionally, Nichols and Stich (2000), other PP theorists have stressed that all typical joint PP scenarios begin with a premise (or premises) that gets the PP started and is the basis by which appropriate thoughts and actions are generated. Similarly, a counterfactual thought emanates from a premise, or what is sometimes termed a conditional statement, for example, ‘What if Tom hadn't called his dad, would Tom be happy or sad?’ (Amsel & Smalley, 2000; Roese & Morrison, 2009). Hence, Weisberg and Gopnik (2013) argue that by adopting a counterfactual premise, the alternative is decoupled from reality.

Furthermore, two versions of the world in mind are held in mind, the current reality, and an alternative version that is decoupled from reality. Representational abuse is avoided by the ability to maintain the reality and imaginary distinctions such that the counterfactual premise does not contaminate what is known to be true in reality, although both real and imaginary representations are held in mind. The question is raised about whether children's errors are an indication of an inability to avoid representational abuse. Weisberg (2013) argues that children's capacity for imagination and reality distinctions are intact; however, various factors like task features, emotional responses, fantasy orientation, etc. may influence performance. This suggests that representational abuse is avoided at each milestone of imaginary thinking and children's struggle with mature CFR may be linked to difficulties with cognitive processing. Amsel and Smalley (2000) have attributed children's difficulty with reasoning from false premises to their having low‐level pretend processing skills in the early years which eventually develop into higher‐level understanding later in development. Specifically, children's low‐level processing appears as a difficulty in remembering both states of affairs in order to reason from the false premise. In other words, although the mechanism for creating alternative worlds in PP and CFR contexts are similar; they are dissimilar in how information about the false state of affairs which could have occurred is used to evaluate the true state of affairs. Amsel and Smalley (2000) suggested that as children develop more powerful cognitive capacities and control systems, more complex forms of CFR become possible. The development of more powerful cognitive capacities is attributed to maturation and control systems but may also have to do with other cognitive skills that develop in tandem with PP and CFR like Executive Functions (EFs).

A criticism, however, is that such a simple unifying framework does not consider how hypothetical event sequences are manipulated to produce contrary‐to‐fact conclusions. For example, the explanation of the cognitive mechanisms of PP and CFR seen in Figure 1 indicates that during PP ‘inferences and other information are *applied* to primary representations quarantined from metarepresentations’, whereas, during CFR ‘intervening processes *change* aspects of the mental representation of the facts to create a second mental representation’. This distinction has been used by Nyhout and Ganea (2019a) to argue that while PP may share processes with CFR, it is not essential to CFR and should be conceptualized separately. According to Nyhout and Ganea (2019a) to reason counterfactually children retrieve from memory a representation of a system of two or more related variables, then manipulate a feature of the representation by positing a false premise and infer causal implications of the false premise. Their key point is that CFR necessarily involves retrieving and manipulating a mental representation of reality that is also in conflict with reality, whereas PP does not. While Nyhout & Ganea emphasized conceptual distinctions between what counts as PP and CFR; their arguments do not nullify that both abilities are in some way dependent on an imaginary representational capacity. It could be that since children's imaginary capacity goes through a period of protracted development, we see the emergence of higher‐order imaginative thinking skills like CFR as children grow older. It may also be that during early development children struggle to change aspects of alternative mental representations because children are inclined to initially stick close to reality in their imagined endeavours (invent, watch and describe imaginary events) as they are still learning about how reality works but when they become secure enough in their real‐world knowledge they can begin to imaginatively explore possibilities that negate their real‐world knowledge (contrast the imaginary alternative with the reality; Harris, 2021; Weisberg, 2013). However, no study has directly tested whether a common underlying imaginary representational capacity is at work. Advanced statistical data analysis techniques like structural equation modelling can be used to identify whether an underlying latent construct could account for associations shared by PP and CFR.

Another view is the claim that PP likely facilitates the skill of CFR since the former precedes the latter (Weisberg & Gopnik, 2013). A few studies have directly explored this position by getting children to reason counterfactually using PP conditions. For instance, Buchsbaum et al. (2012) taught 3–4‐year‐old children a novel causal sequence and then encouraged them to engage in a pretend game to see if they would maintain and act on the causal relation in the context of an imaginary world. The researchers found that children were more successful at answering CFR questions in PP conditions and children's PP and CFR responses were significantly correlated even after controlling for age effects. A related study was repeated with 3–4‐year‐old children from low‐income children in Peru and the USA with similar results – children provided more causally consistent answers in the PP condition (Wente et al., 2022). Earlier work by Dias and Harris (1990) reported that when conditional premises are presented in a make‐believe mode either using a make‐believe intonation, a fantastical setting like another planet, or through visual imagery, children will accept premises that violate their empirical knowledge as a basis for reasoning. These studies show that PP could be an appropriate medium for getting children to begin to practice early CFR abilities.

### Links with executive functions, pretend play and counterfactual reasoning

The cognitive feat of generating imaginary alternatives to reality likely recruits other psychological processes, particularly executive function skills (EFs). EFs are higher‐order, self‐regulatory cognitive processes that aid in the monitoring and control of thought and action (Beck, Carroll, et al., 2011; Beck, Riggs, & Burns, 2011; Garon et al., 2008). EFs is multi‐dimensional construct for which the primary components identified include working memory (WM), inhibitory control, and cognitive flexibility but there are many additional component cognitive skills which are identified as EFs including cognitive skills like planning, self‐regulation, etc. There are arguments for two contrasting views on the developmental trajectory of EFs. One view is that EFs are dissociable in early childhood with inhibition showing improvement during the pre‐school years and less change later on, whereas WM and cognitive flexibility follow a more gradual linear improvement throughout development (Best & Miller, 2010). The contrasting popular view is that EFs follow a protracted developmental trajectory and hangs together as a unitary construct in early childhood up to middle childhood through adolescence when differentiated components are identifiable (Friedman & Miyake, 2017; Reilly et al., 2022). A major implication is whether we are using the most appropriate measures of EFs in studies of PP and CFR.

Several studies have investigated EFs in relation to PP, CFR and the imagination more broadly (Amsel & Smalley, 2000; Beck et al., 2009; Carlson et al., 2014; Carlson & White, 2013; Gautam et al., 2019; Guajardo & Cartwright, 2016). For example, Carlson and White (2013) used a battery of EF tasks to investigate individual differences in EF and pretence representational skills in pre‐school‐aged children. They found a robust, positive correlation which they argued linked EF to the ability to manage dual representations in pretence (Carlson & White, 2013). In a study with 3–4‐year‐olds, Beck et al. (2009) found that inhibition predicted performance on CFR but found no evidence that WM relates to developments in counterfactual thinking ability. The associations between PP, CFR, and inhibition were explored in an experiment by Buchsbaum et al. (2012) and the authors reported that PP and CFR were significantly associated but neither PP nor CFR correlated with inhibition.

The inconsistency in results could arise from task impurities where in addition to the specific EF of interest targeted, non‐executive processes could influence performance on EF tasks (Friedman & Miyake, 2017). Additionally, as EFs may not necessarily be differentiated during early to middle childhood appropriate tasks targeting complex EFs could likely be better at capturing the variance in children's EF abilities at that age. Further research using a battery of EF measures – complex and component, may help clarify associations among PP, CFR, and EFs since all are developing early years skills. At this point, it is unclear if difficulties with CFR are due to increasing demands on EF or simply because CFR is inherently challenging.

### The current study

**FIGURE 2:**
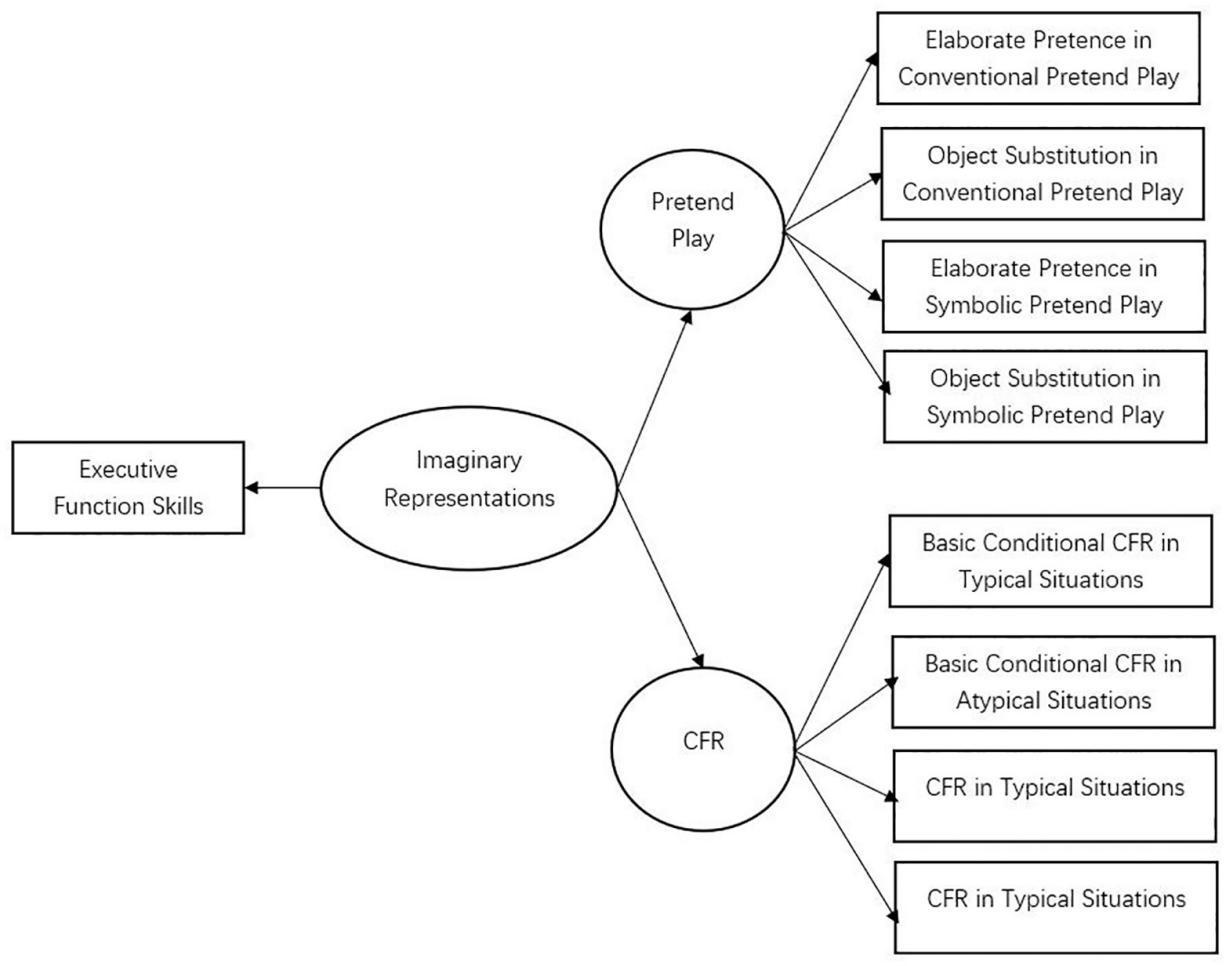
Hypothesized model of pretend play and counterfactual reasoning.

The current study empirically tests whether a latent construct underpins PP and CFR. To do this, we proposed a hypothetical model (see Figure 2) comprising a measurement and structural model that specifies the links between PP and CFR and a second‐order factor that represents the underlying latent construct. This approach uses latent variable modelling – a technique that combines observational data (with a sufficiently large sample size) and theoretical assumptions to begin to draw causal conclusions about variables (Hoyle, 2012). This study is pioneering as it is the first to use a latent variable modelling approach in developmental CFR research.

The measurement model specifies behavioural traits that indicate the various measurement dimensions of the latent construct. We predict that the measurement model will show that the different behavioural traits or measurement dimensions of PP and CFR will cohere as latent constructs. Specifically, behavioural traits of PP are identified from children's ability to engage in object substitution and elaborate pretend play which includes making reference to absent objects as present, verbally attributing properties to objects, and logically sequenced elaborate imaginary actions (Stagnitti, 2007). These measures are assessed in conventional play and symbolic play conditions and capture the variability in children's playful engagements. We predict that all the PP measures are indicators of a broader construct of PP. Likewise, the behavioural traits of CFR represent dimensions relating to basic conditional reasoning and matured CFR in the context of reasoning in typical and atypical situations (Rafetseder & Perner, 2010). Essentially, the task used will elicit children's propensities for basic conditional reasoning and CFR which we predict are indicators of the broad construct of CFR. The measurement model, therefore, should extract latent constructs of PP and CFR from their respective measurement dimensions. Furthermore, the latent constructs should correlate with each other if PP and CFR are related to cognitive skills.

The structural model predicts that a second‐order latent factor will account for unique variance above and beyond associations shared by lower‐order factors (the latent PP and CFR constructs) generated from the measurement model (Chen et al., 2006). The second‐order factor will also be predicted by the measures of EFs. A limitation of this study is that a latent factor of EF cannot be extracted as only two EF measures were included in the study. If the second‐order latent factor is extracted, we posit that it likely represents a general underlying imaginary representational capacity influenced by EF abilities.

In pursuit of testing the hypothetical model, the following research questions will be addressed: (1) Do the dimensions of PP and CFR cohere unto single latent constructs? (2) Do the latent constructs of PP and CFR correlate? (3) Do EFs similarly contribute to unique variance in PP and CFR? (4) Is the hypothetical model of PP and CFR a good fit for the data?

## METHODS

We designed an observational study to examine associations between PP and CFR constructs with children aged 4–5 years old. This age was chosen because it marks when children begin to demonstrate mature CFR (Rafetseder & Perner, 2010, 2014), as well as it being the ‘high season’ of child PP (Kavanaugh, 2006). Institutional ethical approval was provided by the ethics committee at the Faculty of Education, University of Cambridge and the study adhered to standards set by the British Educational Research Association (BERA, 2018). We conducted an a priori check of statistical power using an online sample size calculator for structural equation models to determine the minimum sample needed to detect significant effects in this study (Soper, 2023). The correlation results for PP and CFR (*r50* = .62, *p* < .001) from Buchsbaum et al. (2012) was used posthoc to calculate the statistical power needed to detect an effect using the G*Power calculator resulting in a power value of .95 (Faul et al., 2009). The result from the SEM calculator recommended a minimum sample size of *n* = 156 for a large effect in this study.

### Study participants

The study participants were children aged 4–5 years old. This age group is selected because it is a period where PP is a self‐initiated overt activity before it atrophies by middle childhood, while CFR is burgeoning before it matures by middle childhood. One hundred and ninety‐two children were recruited but participant data were analysed for 189 children (*M* = 4.8 years, range = 4–5.6 years, boys = 101, females = 88) because three participants were autistic so they were excluded from the analysis given the study focused on neurotypical children. The children were recruited from primary schools (*n* = 7) attending Reception (Pre‐Kindergarten) classes. The schools were sampled across Cambridgshire, UK 32% (*n* = 62) of parents reported that children lived in multilingual homes.

### Study procedures

Individual children were engaged in three testing sessions: a PP session, a CFR session and an EFs session. Sessions had a maximum duration of approximately 40 minutes depending on children's interest and engagement with the tasks and were held in a quiet space at the school. PP sessions were video‐recorded and other sessions were audio‐recorded. The three sessions were not administered in any particular order.

### Study measures

All study measures were piloted to check that tasks were appropriate to the target age group and sample as well as to ensure the tasks would be engaging and motivating to children.

#### The Child‐initiated Pretend Play Assessment (CHIPPA)

ChIPPA is a standardised assessment of PP. It was selected because it assesses a child's ability to self‐initiate PP and elicits a range of imaginary pretence thinking skills (Stagnitti, 2007). CHIPPA has two play conditions: conventional‐imaginative play (CV play) and symbolic play (SY play). During each condition, children are engaged in 15 minutes of child‐initiated play. The researcher makes no suggestions of what to play with or how to play but may model play actions midway through the segment. To play, a child sits in front of a ‘Wendy House’ with the researcher. In the CV play condition, the child has presented a commercial playset comprising 1 truck, 1 trailer, 1 male doll, 1 female doll, 1 wrench, 4 sheep, 2 horses, 3 cows, 2 pigs, 3 goats, 1 rooster and 12 fences; and in the SY play condition, unstructured toys including 1 large box, 1 small box, 1 dowel stick, 1 flat stick, 3 pebbles, 1 tin, 1 cone, 1 tea‐towel, 1 face washer and 2 cloth dolls. At the start of each play condition, the child is instructed, ‘Here are some toys for you to play with, you can play with them any way that you like’. CHIPPA reportedly has good concurrent validity, test–retest reliability and inter‐rater reliability (McAloney & Stagnitti, 2009; Stagnitti et al., 2000; Uren & Stagnitti, 2009). CHIPPA is scored by identifying all play actions performed by children– functional play actions, elaborate actions (logically sequenced pretend to play actions) repeated play actions, imitated play actions, reference to absent objects (e.g. the cow is eating the grass) and verbal attributing properties to objects (e.g. the cow is sick) and object substitutions (e.g. the cone is a rocket). In each condition, three subscales are generated: (1) Percentage of Elaborate Play Actions (PEPA) calculated from the raw score for elaborate PP actions over total play actions, (2) counts of the Number of Object Substitutions (NOS) and (3) counts of the Number of Imitated Actions.

#### The Location Change Task

The Location Change Task used was developed by Rafetseder and Perner (2010) and reflects a modified version of a location change task originally developed by Riggs et al. (1998). It was selected because it requires children to answer both basic conditional reasoning (children hold only one alternative in mind) and mature CFR questions (children must hold two alternative locations in mind and inhibit a typical answer). Children are expected to infer the consequence of a protagonist's causal actions as the protagonist moves between typical and atypical locations set up by the researcher. The task was first translated from German to English and the translation was validated by an independent translator to ensure that the content validity was maintained after translation. The task design is based on four story themes relating to four main protagonists: Doctor (props of a hospital, park, swimming pool, doctor, doctor bag, toy‐boy), Teacher (props of a school, house, playground, toy‐teacher, toy‐girl, toy‐students), Fire‐fighter (props of a fire‐station, forest, living room, fire‐extinguisher, fire, toy fire‐fighter) and Police Officer (props of police station, car park, shopping centre, motorcycle, cars, toy police officer).

Each story has four conditions or location changes that involve moving a protagonist between antecedent and consequent locations. The first two conditions involve one location change and the second two conditions involve two location changes. We use the fire‐fighter story to illustrate as all the stories have a similar format. To begin, children are shown the props to develop familiarity with the story locations, for example *firefighter*, *fire station*, *living room* and *forest*. In the first condition (CFR‐T1), the *fire‐fighter* (or protagonists) is moved from a typical antecedent location (*fire station*) to a consequent location (*forest*). The child is asked a memory question, ‘Where is the firefighter now?’ followed by a CFR question, ‘If the fire had not broken out where would the firefighter be?’ and another memory question, ‘where was the firefighter when he received the call that a fire has broken out?’ In the second condition (CFR‐At1), the *firefighter* (or protagonist) is moved from an atypical antecedent location (*living room*) to a consequent location (*forest*). In the third condition (CFR‐T2), the *firefighter* (or protagonist) moves from the atypical antecedent location (*living room*) to the typical antecedent location (*fire station*) before going to the consequent location (*forest*). In the fourth condition (CFR‐AT2), the *firefighter* (or protagonist) moves from the typical antecedent location (*fire station*) to the atypical antecedent location (*living room*) before moving to the consequent location (*forest*). The memory and CFR questions are repeated after each story condition. An overview of the different location changes in each story is detailed in Table 1. The aim was to assess if children can correctly answer whether the protagonist would counterfactually be in the typical or atypical location if he did not go to his last location (Rafetseder & Perner, 2010). The distinction between locations is important as one location change targets basic conditional reasoning and two location changes target mature CFR. Answers to basic conditional reasoning and mature reasoning are the same for typical locations but not atypical locations.

**TABLE 1:** Location changes in the counterfactual reasoning Location Change Task.

CFR stories	Location changes	Typical 1 (CFR‐T1)	Atypical 1 (CFR‐At1)	Typical 2 (CFR‐T2)	Atypical 2 (CFR‐At2)
Doctor Story	First	Hospital (typ location)	Park (atyp location)	Park (atyp location)	Hospital (typ location)
	Second	Swimming pool	Swimming pool	Hospital (typ location)	Park (atyp location)
	Third			Swimming pool	Swimming pool
Teacher Story	First	School (typ location)	Playground (atyp location)	Playground (atyp location)	School (typ location)
	Second	Home of Pupil	Home of pupil	School (typ location)	Playground (atyp location)
	Third			Home of pupil	Home of pupil
Fireman Story	First	Fire station (typ location)	Living room (atyp location)	Living room (atyp location)	Fire station (typ location)
	2nd	Forest	Forest	Fire station (typ location)	Living room (atyp location)
	3rd			Forest	Forest
Police officer Story	1st	Police station (typ location)	Shops (atyp location)	Shops (atyp location)	Police station (typ location)
	2nd	Car park	Car park	Police station (typ location)	Shops (atyp location)
	3rd			Car park	Car park

#### The Head Toes Knees and Shoulders Task (HTKS)

HTKS is a complex measure of EF that taps attentional or cognitive flexibility, working memory and inhibitory control developed by McClelland et al. (2014) for use with pre‐school children. We chose a complex EF measure because we argue that EF is a developing ability in this age group similar to PP and CFR. HTKS involves holding an arbitrary rule in mind, responding according to this rule, and inhibiting a dominant response. HTKS is a short game administered as three sessions using four behavioural rules: ‘touch your head’ and ‘touch your toes’, ‘touch your shoulders’ and ‘touch your knees’. First, children are guided through a series of instructions requiring them to respond naturally but afterwards, the order of performing the rules is switched. HTKS is reported to have good construct validity in relation to other executive function measures (McClelland et al., 2014). Scoring is based on the total number of correct responses for each child.

#### Spin the Pots Task

Spin the Pots is a working memory (WM) task developed by Hughes (1998) and has been used in related studies of alternative thinking (Beck, Carroll, et al., 2011; Beck, Riggs, & Burns, 2011; Hughes & Ensor, 2005). Children are presented with a Lazy‐Susan containing 12 differently shaped boxes each painted a different colour. Ten stickers are hidden in 10 of the boxes. The boxes are covered with a white opaque handkerchief and then the Lazy‐Susan is spun. The child selects one box to find a sticker and returns the box to the Lazy‐Susan. The boxes are covered again, the Lazy‐Susan is spun, and the child gets another try to select a box with a sticker. This continues until the child finds all 10 ten stickers hidden or their 20 chances run out. For each spin, the child needs to remember which boxes have been searched to increase their chances of collecting the 10 stickers in a short time. Scores on the task were calculated as 20 trials minus the total number of errors made.

#### Clinical Evaluation of Language Fundamentals (CELF)

A subset of CELF that assesses children's receptive language skills is used to index the listening and auditory comprehension in children ages 3–6 years (Wigg et al., 2006). Two subtests (a) Sentence Structure and (b) Concepts and Following Directions of CELF were administered. CELF is standardized on a UK sample and reports having good reliability and validity. Each assessment requires participants to respond to 22 sentences and participants receive a score for each correct response.

### Data analyses plans

Preliminary analyses to inspect the data include: check for parametric assumptions, address missing cases, generate descriptive statistics and compute correlations to explore associations among study variables. Four hypotheses were proposed for investigation.

#### Hypothesis 1

We hypothesize that the dimensions of PP and CFR will cohere as single latent constructs, respectively. Based on the argument that children's PP and CFR skills are marked by key dimensions that progress through developmental milestones; we anticipate that the measured dimensions of PP and CFR will be explained by single general constructs, respectively. Confirmatory factor analyses (CFAs) will be conducted to extract the latent constructs of PP and CFR.

#### Hypothesis 2

We hypothesize that the latent construct of PP and CFR will be significantly correlated. Previous research has reported PP and CFR to be significantly correlated, which suggests that the two are related to cognitive skills. A correlation analysis will be undertaken to test this hypothesis.

#### Hypothesis 3

We hypothesize that EFs would account for unique variance in both PP and CFR. Separate studies of PP and CFR have reported that EFs uniquely predict PP and CFR. We predict that if PP and CFR are related cognitive skills, they should have similar patterns of associations with EFs. Separate Hierarchical Multiple Regression (HMR) analyses will be conducted with PP and CFR as dependent variables and EFs as independent variables, controlling for receptive language. The standardized beta (β) values generated from the two HMRs will be compared.

#### Hypothesis 4

We hypothesize that a second‐order latent construct predicted by EFs will account for unique variance, above and beyond that shared, by latent PP and CFR constructs (refer to Figure 2). We predict that the statistical model will support the hypothesized model which suggests that a general underlying imaginary representational (IR) capacity underpins PP and CFR. A second‐order hierarchical structural equation model (SEM) will be run to check the model fit. The Goodness of fit indices are interpreted based on the following guide: the Tucker Lewis Index (TLI) and CFI greater than .90 is a satisfactory fit and greater than .95 is an adequate fit, Standardized Root Mean square (SRMR) and Root Mean Square Error of Approximation (RMSEA) values below .06 indicate adequate fit (Bartholomew et al., 2008; Brown, 2015; Longo et al., 2016). The Bayesian Information Criterion (BIC) index was used to compare the fit of competing models and make a judgement about parsimony (Longo et al., 2016).

### Data preparation

Pretend play was coded from video recordings of CHIPPA. The coding scheme records the child's percentage of elaborate play to total actions (PEPA), the number of object substitutions the child performs (NOS), and if the child imitated any actions modelled by the examiner – number of imitated actions (NIA). PEPA, NOS and NIA were scored for both play conditions such as conventional play (CV play) and symbolic play (SY play; Stagnitti, 2007). A low score for imitated action suggests that the child did not rely on modelled actions for play ideas, whereas a high score for imitated actions might be indicative of developmental delay and or an inability to self‐initiate play ideas. Imitated action scores are excluded from further analysis as they do not represent spontaneous child‐initiated play. A sample of 11% of video data was double coded by the researcher and an experienced coder (the developer of CHIPPA). Inter‐rater reliability was based on intra‐class correlation (ICC) statistics from the mean rating for the two coders (*k* = 2) using a two‐way mixed effects consistency model (see Table 2 below) and resulted in good to excellent reliability across the CHIPPA subscales (Koo & Li, 2016; Shrout & Fleiss, 1979).

**TABLE 2:** CHIPPA inter‐rater reliability results from intra‐class correlations.

CHIPPA sub‐scores	Average ICC	95% CI	Significance level
PEPA Conventional	.88	[0.67, 0.95]	*F* (19, 19) = 7.53, *p* < .001
PEPA Symbolic	.96	[0.90, 0.98]	*F* (19, 19) = 25.03, *p* < .001
NOS Conventional	.90	[0.74, 0.96]	*F* (19, 19) = 9.73, *p* < .001
NOS Symbolic	.96	[0.89, 0.98]	*F* (19, 19) = 23.84, *p* < .001
NIA Conventional	.88	[0.71, 0.95]	*F* (19, 19) = 8.56, *p* < .001
NIA Symbolic	.87	[0.67, 0.95]	*F* (19, 19) = 7.73, *p* < .001

For the CFR task, scores for each correct response to the CFR question are reported for the four conditions (CFR‐T1, CFR‐At1, CFR‐T2, CFR‐At2) across the four counterfactual stories (Doctor, Teacher, Firefighter, Police Officer). Each CFR question has two corresponding memory questions posed before the CFR question (NCQ) and after the CFR question (BCQ). CFR responses are included in the analyses only if both control questions were answered accurately. Subsequent inspection of the data revealed missing cases accounted for less than 5% of CFR tasks, 10% of PP tasks, 2% of receptive language tasks and less than 4% of EF tasks. As the proportion of missing data overall was less than 10%, we used pairwise deletion and full‐information maximum likelihood, as it is generally assumed that missing data below this threshold do not pose a threat to statistical power (Bennett, 2001; Dong & Peng, 2013). The data met assumptions for parametric tests and outliers were retained to maintain the full variability of scores from the different tasks as they were not problematic (Song et al., 2013).

## RESULTS

### Preliminary analyses

#### Descriptive statistics

Data were analysed in Stata (StataCorp, 2019) and descriptive statistics are presented in Table 3. Children had a higher average of elaborate PP scores in the conventional play condition (*M* = 66.76) than in the symbolic PP condition (*M* = 48.57) indicating that children engaged in longer sequences of complex and organized elaborate PP when playing with conventional toys than unstructured toys. Conversely, children had higher averages of NOS‐SY (*M* = 12.86) than NOS‐CV (*M* = 2.25) suggesting that children were less likely to engage in symbolic play when playing with familiar conventional toys like a farm set. NOS‐CV scores had a floor effect and were excluded from further analyses given the limited range of scores.

**TABLE 3:** Descriptive statistics of study variables.

Variable	*N*	Mean	*SD*	Minimum	Maximum
PP conditions					
PEPA_CV	170	66.76	15.77	15	95
PEPA_SY	171	48.57	21.20	0	86
NOS_CV	170	2.25	6.05	0	49
NOS_SY	171	12.86	9.86	0	42
CFR conditions					
NCQ‐T1	184	3.88	0.49	0	4
***CFR‐T1***	***177***	***3.07***	***1.08***	***0***	***4***
BCQ‐T1	184	3.15	1.15	0	4
NCQ‐AT1	184	3.84	0.53	1	4
***CFR‐At1***	***172***	***3.04***	***1.07***	***0***	***4***
BCQ‐AT1	184	3.12	1.21	0	4
NCQ‐T2	183	3.86	0.49	0	4
***CFR‐T2***	***168***	***2.39***	***1.21***	***0***	***4***
BCQ‐T2	183	2.63	1.24	0	4
NCQ‐AT2	182	3.86	.44	1	4
***CFR‐At2***	***166***	***1.97***	***1.20***	***0***	***4***
BCQ‐AT2	182	2.52	1.24	0	4
EFs					
Inhibition	181	27.56	18.15	0	58
WM	185	0.62	0.16	0.35	1
Language					
Receptive language	185	27.27	8.33	7	40

Abbreviations: BCQ‐T1, Before Control Question Typical condition with one location change; CFR_At1, CFR scenarios atypical condition with one location change; CFR_At2, CFR scenarios atypical condition with two location changes; CFR_T1, CFR scenario typical condition with one location change; CFR_T2, CFR scenarios typical condition with two location changes; EFs, Executive Functions; NCQ‐T1, Now Control Question Typical condition with one location change; NOS‐CV, Number of object substitutions in the conventional play condition; NOS‐SY, Number of object substitutions in the symbolic play condition; PEPA‐CV, Percentage of elaborate PP in the conventional‐imaginative play condition; PEPA‐SY, Percentage of elaborate PP in the symbolic play condition; WM, Working Memory.

Children performed better in CFR conditions with one location change that is instances when the protagonist (e.g. *firefighter*) moved from a typical location (*fire station*) to a consequent location (*forest*) – CFR‐T1 = 77% (*M* = 3.07, *SD* = 1.08) and from an atypical location (*living room*) to a consequent location (*forest*) – CFR‐At1 = 76% (*M* = 3.04, *SD* = 1.07). In comparison, children had lower scores for CFR questions in conditions with two location changes, that is instances when the protagonist (e.g. *firefighter*) moved from an atypical location (*living room*) to a typical location (*fire station*) to the consequent location (*forest*) – CFR‐T2 = 60% (*M* = 2.39, *SD* = 1.21) and from a typical location (*fire station*) to an atypical location (*living room*) to the consequent location (*forest*) – CFR‐At2 = 49% (*M* = 1.97, *SD* = 1.20).

#### Correlations

Pairwise Pearson correlation was used to investigate the associations among the study measures (see Table 4). All PP traits were significantly correlated with each other, and all CFR traits were significantly and moderately correlated with each other. The complex EF measure (HTKS) was significantly correlated with all variables. In contrast, WM shared non‐significant negative correlations with most study variables, including HTKS (*r* = −.20, *p* = .006). Receptive language shared significant correlations with all study variables except NOS‐SY.

**TABLE 4:** Correlation matrix for study variables.

Variables	Pretend play			Counterfactual reasoning				EFs		Language
	PEPA‐CV	PEPA‐SY	NOS‐SY	CFR‐T1	CFR‐AT1	CFR‐T2	CFR‐AT2	HTKS	WM	
PEPA‐CV	–									
PEPA‐SY	.50*	–								
NOS‐SY	.20*	.42*	–							
CFR‐T1	.27*	.27*	.14	–						
CFR‐AT1	.24*	.33*	.25*	.64*	–					
CFR‐T2	.27*	.26*	.24*	.44*	.54*	–				
CFR‐AT2	.14	.23*	.35*	.44*	.48*	.39*	–			
HTKS	.24*	.27*	.17*	.42*	.32*	.31*	.34*	–		
WM	‐.09	−.06	.04	−.07	−.13	−.19*	−.05	−.20*	–	
Language	.22*	.29*	.14	.48*	.39*	.38*	.35*	.54*	−.23*	–

*Note*: *p = .05.

Abbreviations: CFR_At1, CFR scenarios atypical condition with one location change; CFR_At2, CFR scenarios atypical condition with two location changes; CFR_T1, CFR scenarios typical condition with one location change; CFR_T2, CFR scenarios typical condition with two location changes; HTKS, Head, Toes, Knees and Shoulders Task of Complex EF; NOS‐CV, Number of object substitutions in the conventional play condition; NOS‐SY, Number of object substitutions in the symbolic play condition; PEPA‐CV, Percentage of elaborate PP in the conventional‐imaginative play condition; PEPA‐SY, Percentage of elaborate PP in the symbolic play condition; WM, Working Memory.

### RQ1: Do the dimensions of PP and CFR cohere unto latent single constructs?

**FIGURE 3:**
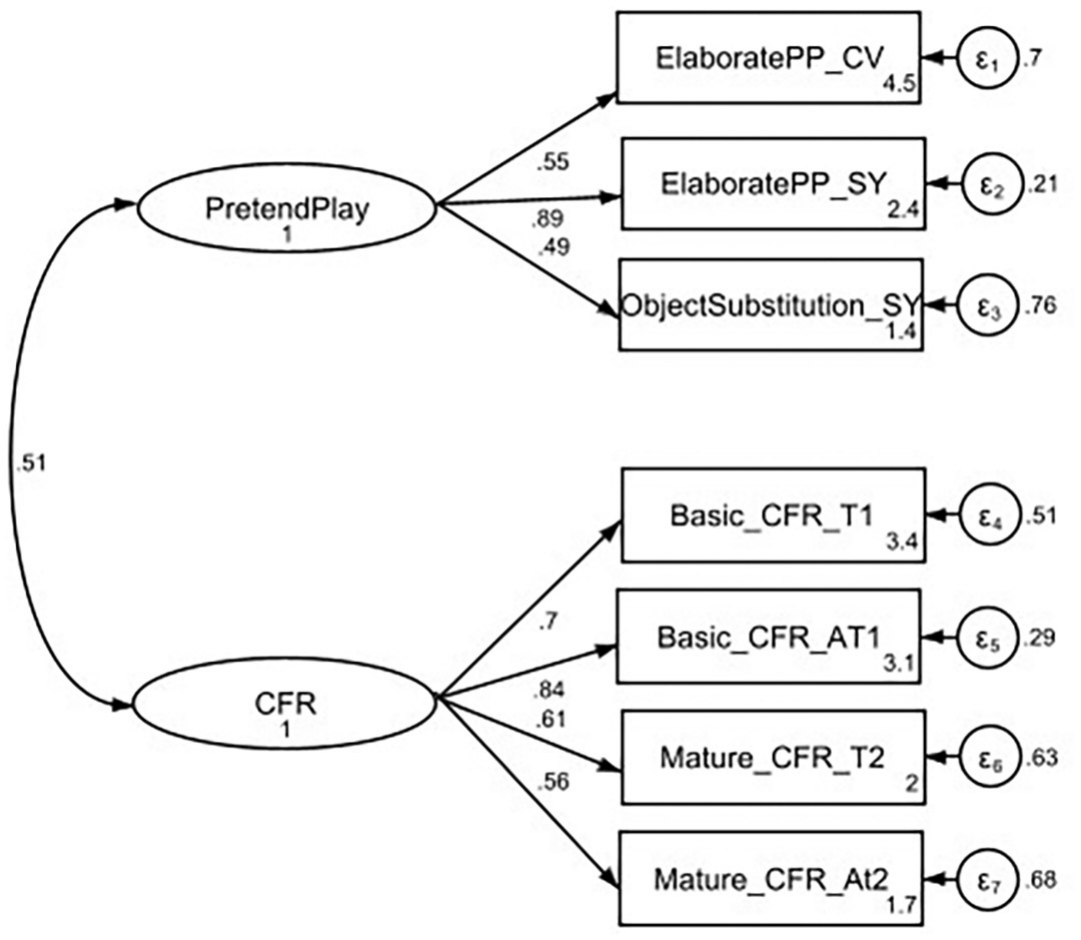
CFA – two‐factor model PP and CFR.

The results of the two‐factor measurement model comprising PP (PEPA‐CV, PEPA‐SY, NOS‐CV) and CFR (T1, At1, T2, At2) dimensions showed the model was a good fit for the data (see Figure 3). The dimensions of PP and CFR cohered as single latent factors for each construct. The model chi‐square was significant *χ* ^2^(13) = 27.49, *p* = .01 and a chi‐square degrees of freedom ratio of 2.11 indicates a satisfactory fit. The other fit indices: a TLI of .91, a CFI of .94, an SRMR of .06 indicate a satisfactory fit for the two‐factor model. The RMSEA recommended cut‐off values less than .08 and the result in this study is .08. Taken together, the combination of fit indices is satisfactory without modifications. All items had significant standardized estimates ranging from *β* = .49 to *β* = .89 which are acceptable as Kline (2015) suggests factor loadings lower than .45 are indicators of unacceptable fit. The individual domains had acceptable internal consistency (Cronbach alpha): *α* = .63 for the PP domain and *α* = .77 for the CFR domain.

### RQ2: Do the latent constructs of PP and CFR correlate?

The latent constructs, PP and CFR, were significantly correlated with each other, *r* = .51, *p* = .001 even after controlling for age.

### RQ3: Do EFs similarly contribute to unique variance in PP and CFR?

The latent factors scores predicted for PP and CFR were used in separate Hierarchical Multiple Regressions (HMR) as dependent variables. Age in months and gender were entered in the first block, receptive language was entered in the second block, and inhibition and working memory were entered in the third block. Each HMR model examined whether the independent variables accounted for unique variance, above age and gender in PP or CFR and the two HMR models were compared (see Table 5).

**TABLE 5:** HMR: Contribution of age, receptive language and EFs to PP and CFR.

	PP‐dependent variable			CFR‐dependent variable		
	*β*	*R* ^2^	∆*R* ^2^	*Β*	*R* ^2^	∆*R* ^2^
Step 1		.07			.05	
Constant						
Age	.17*			.21*		
Gender	.21*			.10		
Step 2		.13	.06*		.23	.18*
Constant						
Age	.11			.12		
Gender	.19*			.08		
Receptive language	.28*			.43*		
Step 3		.16	.03**		.26	.03*
Constant						
Age	.07			.07		
Gender	.19*			.07		
Receptive language	.16			.32*		
Complex EF	.21*			.22*		
Working memory	−.03			−.01		

*Note*: *p 〈 .05; **p 〈 .001

For the HMR with PP as a dependent variable, each block of the model was significant and accounted for 7%, 13% and 16% of the variance in PP, respectively. The first block comprised age and gender *F*(2, 175) = 6.66, *p* = .002 and both were significant predictors of PP; age (*β* = .17, *p* = .02) and gender (*β* = .21, *p* = .01). In the second block, receptive language was added *F*(3, 174) = 8.99, *p* = .001 and gender (*β* = .19, *p* = .01) and receptive language (*β* = .26, *p* = .001) were significant predictors. Finally, in the third block, inhibition and WM were added *F*(5, 172) = 6.73, *p* = .001 but only gender (*β* = .19, *p* = .01) and inhibition (*β* = .21, *p* = .02) were retained as significant predictors. The change in r^2^ in this final block had a positive trend (*p* = .05).

For the HMR with CFR as a dependent variable, each block of the model was significant and accounted for 5%, 23% and 26% of the variance in CFR, respectively. The first block comprised age and gender *F*(2, 175) = 5.06, *p* = .01 and only age was a significant predictor; age (*β* = .21, *p* = .01). In the second block, receptive language was added *F*(3, 174) = 17.45, *p* = .001 and was the only significant predictor (*β* = .43, *p* = .001). Finally, in the third block, inhibition and WM were added *F*(5, 172) = 12:31, *p* = .001 but only language (*β* = .32, *p* = .001) and inhibition (*β* = .22, *p* = .01) were retained as significant predictors. The change in r‐square in this final block was significant (*p* = .03). When the two HMRs are compared, inhibition remains a consistently significant predictor of both PP and CFR.

### RQ4: Is the hypothetical model of PP and CFR a good fit for the data?

**FIGURE 4:**
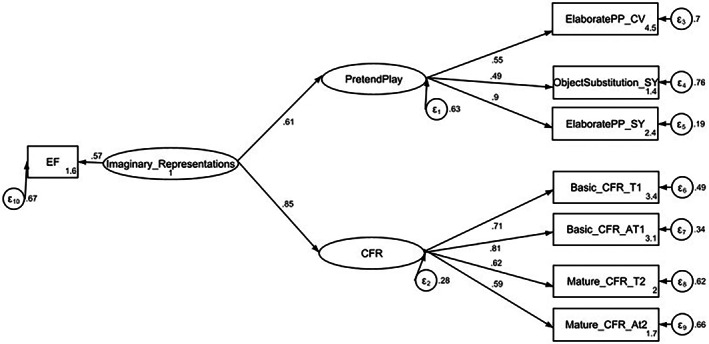
SEM‐Hierarchical second‐order factor model of PP and CFR.

An SEM examined the extent to which the hypothetical model which delineated links between PP, CFR, and EFs (specifically inhibition) was a good fit for the data. The second‐order hierarchical model revealed that a common underlying factor, accounted for the shared associations between PP and CFR and this factor was predicted by inhibition (see Figure 4). We think this factor represents an imaginary representational (IR) capacity. The model chi‐square was significant *χ* ^2^(18) = 32.05, *p* = .02 and the chi‐square degrees of freedom ratio of 1.78 indicates an adequate fit. The combination of other fit indices: TLI of .92, a CFI of .5, an SRMR of .06, and an RMSEA of .07 indicates a satisfactory fit without modifications. We did not optimize the data for fit once the hypothesis was supported as we were interested in the most parsimonious fit of the hypothetical model tested.

## DISCUSSION

The mechanism by which the imagination facilitates ubiquitous cognitive abilities like PP and CFR is not always clear although it is central to thinking about alternative versions of reality. In this study, we tested four hypotheses related to PP, CFR and the influential role of EFs to advance our understanding of the cognitive architecture of the imagination. We discuss several notable findings from this work and consider the implications of the results.

The first notable finding is that from the respective measured dimensions of PP and CFR single unitary latent constructs were extracted. This is interesting because both PP and CFR are multi‐dimensional constructs comprising various cognitive indicators that signal early development. The results showed that the dimensions of PP and CFR hang together as indicators of each construct in this study. The dimensions of PP in this study comprised behaviours like object substitution, referring to absent objects as present, verbally attributing properties to objects, and logically sequenced elaborate imaginary actions (Stagnitti, 2007). The measured dimensions compare with the first two stages of Thompson and Goldstein (2019) proposal of how PP develops – object substitutions, attribution of pretend properties, social interactions within pretend, role enactment and pretence‐related metacommunication and should be measured in developmental research.

Moreover, this is the first study to find evidence of a general construct of CFR. The task considers both basic condition reasoning and mature CFR responses and similar to Rafetseder and Perner (2010), the results show that children are less successful at mature CFR where they had to hold two location changes in mind (CFR‐T2 = 60% and CFR‐AT2 = 49%) as opposed to basic conditional reasoning where only one location change needs to be remembered (CFR‐T1 = 77%, CFR‐AT1 = 76%). In contrast to Rafetseder and Perner who argue a distinction between basic conditional reasoning and CFR, we suggest that the result of the CFA supports Beck, Riggs, and Burns (2011) view that CFR is not one critical development but comprises progressive developmental milestones.

The second hypothesis that PP and CFR are related to cognitive skills was supported. PP and CFR were significantly positively correlated (*r* = .51) at a latent level as well as a measurement level. Similar positive correlations between PP and CFR are reported by Buchsbaum et al. (2012) who reported significant correlations (*r* = .59) after controlling for age in one experiment and after controlling for age, EF and conservation in a second experiment (*r* = .38). To the best of our knowledge, no other study has investigated the association between PP and CFR at a structural level.

The third hypothesis predicted EFs would uniquely contribute to PP and CFR. The rationale for this hypothesis is that if PP and CFR share cognitive processes then they should have similar patterns of associations with other cognitive skills. We used two measures of EF, a complex EF measure of WM, inhibition and cognitive flexibility (McClelland et al., 2014) and the second was a component measure of WM (Hughes, 1998). Complex EFs similarly accounted for significant unique variance in PP (*β* = 21) and CFR (*β* = 22) over and above age and receptive language but WM did not. Inconsistent associations between WM, PP, and CFR are reported in the literature. For example, Carlson et al. (2014) reported that WM did not significantly predict child engagement in PP after controlling for factors like age and language. Beck et al. (2009) also reported no significant correlations between WM and CFR. In contrast, Guajardo and Cartwright (2016) found that WM significantly predicted performance in CFR, and Drayton et al. (2011) reported WM, inhibition and CFR were all significantly correlated. Previous studies have reported significant links between other EF components like inhibition with PP and CFR. For instance, Carlson et al. (2014) reported positive correlations between inhibition and the ability to manage dual representations in PP, and Beck et al. (2009) reported positive correlations between inhibition and CFR. However, a component inhibition measure was not used in this study.

Moreover, the measure of complex EF used in this study could also be described as a general EF measure which suggests that when treated as a broad unitary construct with pre‐schoolers, EF is integral to both PP and CFR. It may be that EFs are not well differentiated during early childhood (Reilly et al., 2022) and the use of a general measure that concurrently taps multiple EFs – WM, inhibition and cognitive flexibility, is better at capturing the imaginative thinking skills used during PP and CFR. These EF skills become even more important during CFR when children need to perform the challenging skill of changing only the necessary features imported into the imagination by holding all else constant and inhibiting the prepotent tendency to defer to general real‐world knowledge (Nyhout & Ganea, 2019a, 2019b). We think that since PP, CFR, and EFs follow protracted development during the early years; future studies should aim to use measures that capture these cognitive abilities as developing skills.

Fourthly, the findings supported the hypothetical model that a second‐order latent factor predicted by EF accounts for unique variance above and beyond that shared by the latent constructs PP and CFR. We suggest that the second‐order factor likely represents a general capacity for imaginary representations. The findings from this study support Weisberg and Gopnik's (2013) unifying framework of imaginative processes. Essentially, given that PP and CFR both involve alternative imaginative thinking about reality; they likely share underlying cognitive processes – a common imaginary capacity which recruits EFs. Future work can explore if any other similar cognitive skills are also recruited.

A similar paradigm is described by Amsel and Smalley (2000) who proposed a model of CFR about possibilities to depict how interactions between true and false states of affairs are likely reliant on the ability to imagine possible worlds. They describe a process where (a) the representational format of the true world and its counterfactual representation, in any given context, operate through a process whereby a sequence of events from the real world are copied and then edited by altering a specific event, to result in the final change being the alternative (imaginary) representation; and (b) the processing requirements of the edited imagined representation involves comparing and contrasting to the real‐world representation to reason from the false premise (Amsel & Smalley, 2000). However, we argue that for this model to be upheld, it would need to account for (a) differences in imaginary ideas that are derived from pretending or CFR; (b) the discrepancies in the demands that PP and CFR may place on the imagination, that is pretending is typically open‐ended, whilst CFR is more constrained and (c) the incremental changes in child PP or CFR across the lifespan, for example pretending to emerge as object substitutions to social PP or progressing from basic conditional reasoning to mature CFR. Furthermore, we infer that the notion of an underlying imaginative representation should extend to other forms of hypothetical thinking about alternatives to reality, for example future hypotheticals, syllogistic counterfactuals, false belief, etc., and other indicators of PP, for example, social interactions within pretend, role enactment and pretence‐related metacommunication. Future research could test new hypothetical models that include the other forms of alternative thinking about reality as additional variables to structurally test if a similar second‐order factor would be extracted. Other advanced statistical models like bifactor models could assess the robustness of the structural model by testing if a general latent construct (imaginary capacity) that is independent of the domain‐specific constructs (PP, CFR, FB, etc.) could also fit the data (Chen et al., 2006).

### Limitations and future directions

Data for this study were collected at one time point; hence, we are not able to infer causality in this study (Goodwin & Goodwin, 2014). Limits on testing time also impacted the number of indicators that can be included in the measurement model. Future attempts at testing the structural relation between PP and CFR could more rigorously test individual EF components and additional indicators of CFR and PP to increase the robustness of the structural model (Kline, 2015). The study is generalizable to the extent that it applies to similar‐age children living in relatively affluent communities.

## CONCLUSION

This study empirically tested the role of the imagination in a unifying framework of PP, CFR and EF. The study resulted in several notable findings including (a) robust support for viewing PP and CFR as multi‐dimensional constructs; (b) evidence that PP and CFR are significantly correlated; (c) evidence that EFs account for similar variance in PP and CFR and (d) support for the hypothesis that PP and CFR abilities draw on a general underlying imaginary thinking capacity influenced by EF skills. Put together, the study shows that although the broad cognitive capacity for imaginative thinking comprises different forms of thinking about alternatives to reality; they are related early years developmental thinking skills.

## AUTHOR CONTRIBUTIONS

**Gill Althia Francis:** Conceptualization; data curation; formal analysis; funding acquisition; investigation; methodology; project administration; resources; software; validation; visualization; writing – original draft; writing – review and editing. **Jenny Louise Gibson:** Conceptualization; funding acquisition; methodology; supervision; writing – review and editing.

## FUNDING INFORMATION

This research was funded by the Research Centre for Play Education Development and Learning (PEDAL) and ESRC WRDTP Postdoctoral Grant funding #R23162.

## CONFLICT OF INTEREST STATEMENT

The authors have no conflict of interest to declare.

## Data Availability

Data are available on request only due to the ethical permissions granted.
